# The efficacy of tonsillectomy in the management of PFAPA syndrome: a systematic review and meta-analysis

**DOI:** 10.3389/fsurg.2026.1825542

**Published:** 2026-07-20

**Authors:** Mingjiang Xia, Yan Chen, Yingchao Yang, Yao Wang, Yinghong Zhang, Jingyi Chen, Kaiming Su

**Affiliations:** 1 Department of Otorhinolaryngology Head and Neck Surgery, Shanghai Sixth People's Hospital Affiliated to Shanghai Jiao Tong University School of Medicine, Shanghai, China; 2 Shanghai Key Laboratory of Sleep Disordered Breathing, Shanghai Sixth People's Hospital, Shanghai, China; 3 Pediatric Obstructive Sleep Apnea Center of Shanghai Sixth People's Hospital, Shanghai, China

**Keywords:** pediatric autoinflammatory disease, PFAPA syndrome, postoperative remission, systematic review, tonsillectomy

## Abstract

**Objective:**

To evaluate tonsillectomy's effectiveness in treating PFAPA syndrome through a systematic review and meta-analysis, providing evidence to guide clinical decisions and improve patient outcomes.

**Data source:**

The PubMed, Cochrane Library, and Google Scholar databases, covering the period from their inception up to October 2024.

**Methods:**

A meta-analysis of randomized controlled trials comparing surgical vs. conservative treatment was conducted to evaluate the postoperative complete remission rate, duration of fever, and frequency of fever episodes. Binary outcomes were analyzed using odds ratios (OR) and continuous outcomes using mean differences (MD) with 95% confidence intervals. The DerSimonian-Laird random-effects model was used, with the Hartung-Knapp-Sidik-Jonkman (HKSJ) adjustment, prediction intervals, and leave-one-out analysis as sensitivity analyses to address the small number of included studies.

**Results:**

Three randomized controlled trials (81 patients) met the inclusion criteria. Tonsillectomy significantly improved postoperative complete remission rates (pooled OR = 42.21, 95% CI: 9.34–190.74, *P* < 0.0001, I² = 0%), a finding confirmed by HKSJ sensitivity analysis (OR = 42.21, 95% CI: 2.93–609.09, *P* = 0.026) and robust in leave-one-out analysis. The pooled mean difference in fever duration was −3.15 days (95% CI: −6.78 to 0.49, *P* = 0.090), which was not statistically significant, with substantial heterogeneity (I² = 68.5%). Surgery significantly reduced fever episode frequency (MD = −0.41 episodes/person-month, 95% CI: −0.66 to −0.15, *P* = 0.002, I² = 0%). No postoperative complications were reported.

**Conclusion:**

This meta-analysis suggests that tonsillectomy can provide significant benefits for children with PFAPA, particularly in achieving complete remission and reducing episode frequency. However, evidence on fever duration remains inconclusive, whereas the reduction in episode frequency was statistically robust. Given the small number of included studies, these results should be considered hypothesis-generating. Future large-scale, well-designed RCTs are needed to confirm these findings.

## Introduction

PFAPA syndrome (Periodic Fever, Aphthous Stomatitis, Pharyngitis, and Adenitis) is a self-limiting condition that primarily affects children (1). Although its exact etiology remains incompletely understood, the characteristic symptoms significantly impact the quality of life for affected children and their families (2, 3). PFAPA typically manifests in early childhood and is diagnosed based on clinical history, which includes recurrent febrile episodes exceeding 39.8 °C lasting 3–6 days and recurring every 3–8 weeks, with relatively mild symptoms between episodes (4, 5). However, these frequent attacks not only increase the utilization of medical resources but also impose substantial psychological and economic burdens on patients and their families.

Currently, treatment modalities for PFAPA primarily encompass conservative management, pharmacotherapy, and surgical intervention (6–8). Conservative treatment is generally appropriate for patients with mild symptoms or those who prefer less aggressive approaches, focusing primarily on symptomatic relief. Pharmacological treatments, such as corticosteroids and nonsteroidal anti-inflammatory drugs (NSAIDs), can mitigate episodes to some extent but are associated with interindividual variability and potential adverse effects, including shortened intervals between febrile episodes, insomnia, and behavioral disturbances (9, 10). Additionally, concerns about the long-term systemic side effects of medications among some parents can negatively impact treatment adherence.

In recent years, tonsillectomy has garnered increasing attention as a potential surgical treatment option for PFAPA. Early studies evaluating its efficacy have yielded mixed results; some indicate that tonsillectomy can significantly reduce the frequency and severity of attacks and even achieve symptom remission (11, 12), while others have not clearly demonstrated its advantages (8). In 2019, the American Academy of Otolaryngology–Head and Neck Surgery (AAO-HNS) updated its clinical practice guidelines, in which tonsillectomy was listed as an option (rather than a strong recommendation) that clinicians may consider for children with PFAPA who meet certain criteria, based on limited available evidence (13).

Given the heterogeneous findings of existing research and the potential therapeutic value of tonsillectomy in PFAPA management, this study aims to conduct a systematic review and meta-analysis of the current literature to comprehensively evaluate the overall impact of tonsillectomy on treatment outcomes for PFAPA patients. By quantitatively analyzing data from various studies, this research intends to provide a more robust evidence base for clinical decision-making, optimize treatment strategies for PFAPA, and ultimately enhance the quality of life for affected patients.

## Eligibility standard

The inclusion criteria for literature in this study were defined using the PICOS framework as follows: Patients (P) were children diagnosed with PFAPA; Interventions (I) included intracapsular tonsillectomy (TT) and tonsillectomy (TE); Comparators (C) were conservative treatments (Control); Outcomes (O) encompassed cure rates, duration of symptoms, and number of recurrences; Study designs (S) included randomized controlled trials (14). The exclusion criteria were: inability to access the full text, inability to extract valid data, literature not addressing the aforementioned outcome measures, study designs other than two-arm trials, non-English language publications (this restriction was applied due to resource constraints and is acknowledged as a potential source of language bias in the Limitations section), editorials, reviews, meta-analyses, case reports, conference abstracts, as well as studies with low confidence after screening and those published after October 2024.

## Data source, information search and study selection

**Figure 1:**
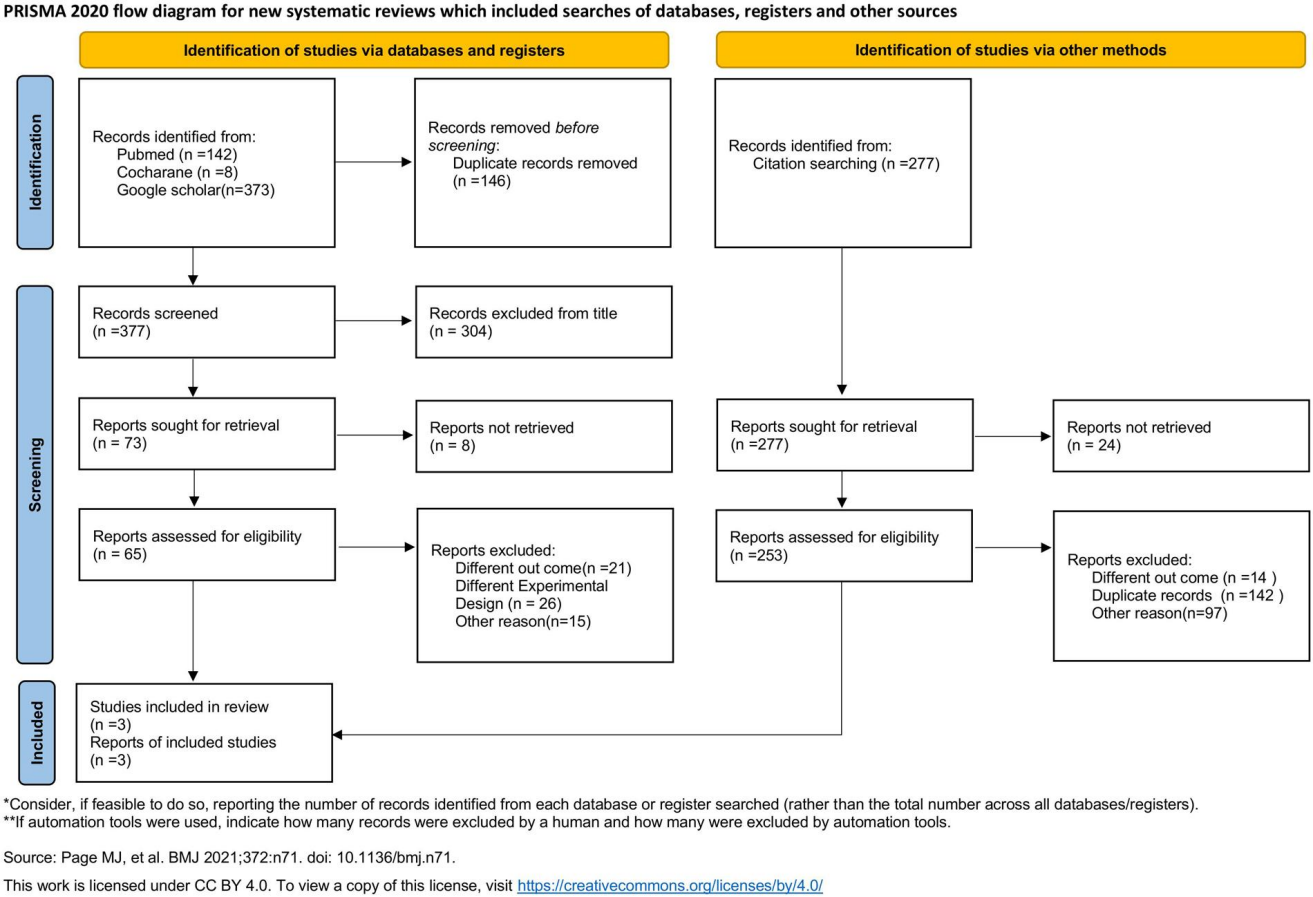
Flow diagram of the trials identified and selected.

This systematic review was conducted and reported in accordance with the Preferred Reporting Items for Systematic Reviews and Meta-Analyses (PRISMA) 2020 guidelines. The completed PRISMA checklist is provided as Supplementary Material S1. Literature searches were performed in PubMed, Cochrane Library (CENTRAL), and Google Scholar from database inception through October 31, 2024. The search strategy for PubMed was: (“PFAPA” OR “periodic fever, aphthous stomatitis, pharyngitis, and adenitis” OR “periodic fever syndrome”) AND (“tonsillectomy” OR “tonsillotomy” OR “adenotonsillectomy” OR “tonsil surgery”). Equivalent search terms were adapted for Cochrane Library and Google Scholar. No date or language filters were applied during the electronic search; however, non-English publications were subsequently excluded during screening (see Exclusion Criteria). The reference lists of all included studies and relevant reviews were manually screened to identify any additional eligible studies. All screening steps were performed independently by two researchers(MX and YC). The screening process involved four steps and three rounds: deduplication, title screening, abstract screening, and full-text screening (Figure 1). In cases of disagreement, the conflicting results were submitted to a senior reviewer for resolution. The final screening outcomes were documented and recorded in a structured table.

## Assessment of risk of bias and strength of evidence

**Figure 2:**
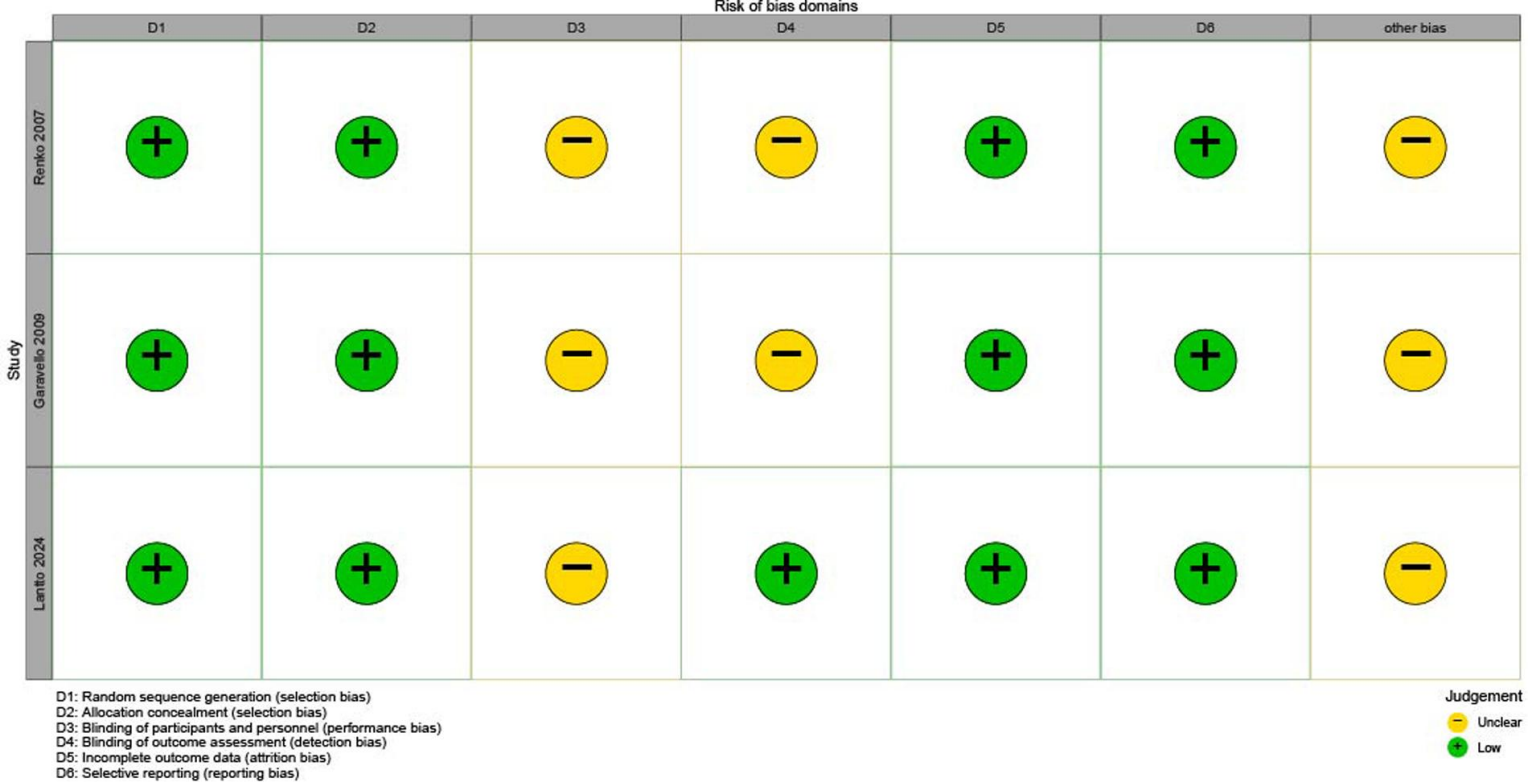
Summary of the quality assessment of the included studies.

The methodological quality of included studies was assessed using the original Cochrane Risk of Bias tool (RoB 1.0) (15), which evaluates seven domains: random sequence generation (selection bias), allocation concealment (selection bias), blinding of participants and personnel (performance bias), blinding of outcome assessment (detection bias), incomplete outcome data (attrition bias), selective reporting (reporting bias), and other sources of bias. Each domain was judged as “low risk,” “high risk,” or “unclear risk” of bias. Two researchers (MX and YC) independently assessed each study, and disagreements were resolved by discussion with a senior reviewer (KS). A pre-specified criterion was established whereby studies judged as high risk of bias in more than four of the seven domains would be excluded; however, no studies were excluded on this basis, as all three included RCTs demonstrated acceptable overall quality (Figure 2).

## Data collection and analysis

During data collection, two independent researchers extracted the following information: PMID, first author, publication date, journal name, basic participant characteristics (including sample size, gender, and age), intervention measures, and the outcome variables included in the studies (cure rate, frequency of attacks, and duration of each attack). Cure rates were represented as binary variables, while attack frequency was measured as the number of attacks per person per month. For studies reporting only the total number of attacks, the attack frequency was calculated using the follow-up duration before including the data in the meta-analysis. Where numerical data were not directly reported, values were estimated from published figures (accurate to one decimal place) or derived from reported summary statistics using appropriate formulas (e.g., Poisson-based standard deviation estimation). All instances of data estimation are noted in Table 1.

**Table 1:** Summary table of literature of PFAPA.

Year	Type	Group	Patients	Male	Age	Remission rate	Relief time	Fever episodes	Duration of symptoms	Medication	Follow-up time
								(Mean, SD, episodes/person/month)	(Mean, SD, days/person/month)		
Renko et al. 2007	RCT	TE	14	8	4.2	100%	NA	0.05, 0.59	NA	Permitted	6 months
		Control	12	8	4	50%		0.44, 0.4		Permitted	6 months
Garavello 2009	RCT	ATE	19	10	5.4	63%	18.6 weeks	0.1, 0.32	1.7, 0.5	Permitted	18 months
		Control	20	13	4.9	5%	7.5 weeks	0.52, 0.72	3.5, 0.75	Permitted	18 months
Lantto 2024	RCT	TT	8	4	4.5	88%	NA	NA	0.8, 1.4	Prohibited	9 months
		Control	8	6	3.9	25%			6.5, 6	Prohibited	9 months

Fever Episodes (Mean, SD, times/person/month), Duration of Symptoms (Mean, SD, days/person/month).

TE, tonsillectomy; TT,  tonsillotomy; ATE, adenotonsillectomy; RCT, randomized controlled trial; SD, standard deviation; NA, not available.

For studies with unclear standard deviations in attack counts, a Poisson distribution-based formula: $SD\;=\;\sqrt{\bar{X}}$ (X represents the average attack frequency) (16). Meta-analyses were performed using R version 4.4.1 with the “meta” and “metafor” packages. A meta-analysis of randomized controlled trials comparing surgical vs. conservative treatment was conducted to evaluate three pre-specified outcomes: (1) complete remission, defined as the complete absence of PFAPA episodes during the entire post-operative follow-up period as reported by each original trial; (2) episode frequency, defined as the mean number of PFAPA episodes per person per month during follow-up; and (3) fever duration, defined as the cumulative symptomatic days per person per month. Binary outcomes (cure rates) were analyzed using odds ratios (OR) with 95% confidence intervals (CIs), while continuous outcomes (fever duration and attack frequency) were analyzed using mean differences (MD) with 95% CIs. The between-study variance *τ*² was estimated using the DerSimonian-Laird (DL) random-effects model. Given the small number of included studies (*n* = 3), several additional measures were implemented to enhance analytical robustness, as recommended by Zhou and Shen (17). First, the Hartung-Knapp-Sidik-Jonkman (HKSJ) adjustment was applied as the primary sensitivity analysis, as the standard DL method may produce overly narrow confidence intervals when the number of studies is limited, leading to inflated Type I error rates (18). The HKSJ method employs a t-distribution with k−1 degrees of freedom and a refined variance estimator that accounts for the uncertainty in *τ*² estimation, producing more conservative and appropriate inferences (19). Second, 95% prediction intervals were calculated to estimate the range of true effects expected in future studies, providing a more clinically meaningful representation of between-study heterogeneity. Third, a leave-one-out sensitivity analysis was performed to assess whether the overall conclusions were driven by any individual study. Heterogeneity among studies was assessed using the I² statistic and Cochran's *Q* test; however, the interpretation of I² was made with caution, as this statistic has been shown to be unreliable in meta-analyses involving few studies. An I² value greater than 75% indicated substantial heterogeneity, prompting the selection of a random-effects model. Although the three included studies employed different surgical techniques—tonsillectomy alone (Renko 2007), adenotonsillectomy (Garavello 2009), and tonsillotomy via Coblation (Lantto 2024)—we considered pooling to be justified for the following reasons: (1) all three procedures involve the removal or substantial reduction of tonsillar tissue, which is the hypothesized immunological mechanism underlying symptom resolution in PFAPA; (2) the primary outcome (complete remission) reflects a clinically unambiguous endpoint that is unlikely to be differentially affected by the extent of tonsillar tissue removal; and (3) the extremely small number of available RCTs (*n* = 3) precludes meaningful subgroup or network meta-analysis by surgical technique. Nevertheless, the heterogeneity in surgical approach represents a significant source of clinical heterogeneity, and caution is warranted in generalizing these pooled estimates to any single surgical technique. Due to the limited number of included studies (<10), formal assessment of publication bias (e.g., funnel plot or Egger's test) was not performed, as such tests have low statistical power when fewer than 10 studies are available.

## Results

### Search results

A total of 277 studies were identified through database searches, and additional searches were conducted using the reference lists of these studies. In the first phase of screening, non-English language articles, reviews, reports, replies, and conference-type publications were excluded based on their titles. In the second phase, screening was conducted according to the PICOS inclusion criteria, with further evaluation of abstracts and full texts. Studies were excluded if they involved highly heterogeneous study populations, did not meet the required study design standards, lacked outcome data, or if the full text could not be obtained. The detailed screening process is illustrated in Figure 1. Ultimately, 274 studies were excluded, and 3 studies met the inclusion criteria (20–22).

These three randomized controlled trials included a total of 81 patients, of whom 39 were male (48%). The average age of the enrolled patients ranged from 3.0 to 5.4 years, with the youngest patient aged 1.5 years and the oldest 14 years (based on data from 2007). The follow-up periods ranged from 6 to 18 months. All three studies examined the impact of surgical intervention on PFAPA patients. Specifically, all three studies assessed complete postoperative remission rates, two studies evaluated postoperative fever duration (2009 and 2024), and two studies investigated the average frequency of fever episodes after surgery. The basic characteristics of these studies are presented in Table 1.

### Method

In the study by Renko 2007, participants were randomly assigned to either a surgical group or a control group. Those in the surgical group underwent tonsillectomy within one month, while the control group received no initial treatment.

In Garavello 2009, patients in the surgical group underwent a standardized adenoidectomy and tonsillectomy (adenotonsillectomy) with uniform procedures to ensure consistent intervention. Patients in the control group received no surgical intervention or other treatments, serving as an observation-only group.

In Lantto 2024, participants were divided into a surgical group and a control group. The surgical group underwent partial tonsil removal using Coblation® radiofrequency ablation technology (Smith & Nephew, Hertfordshire, UK). During the procedure, the surgeon visually and manually assessed the size of the tonsils, carefully excising at least half of the palatine tonsil while preserving the tonsillar capsule and performing no intervention on the adenoids. The control group received no treatment and was only followed for observation.

### Patients

In Renko 2007, 26 Caucasian children were enrolled. The intervention group included 14 children with a mean age of 4.2 years, and the control group had 12 children with a mean age of 4.0 years. All participants met the diagnostic criteria for PFAPA syndrome: at least five episodes of unexplained, recurrent high fevers (≥38.5 °C) occurring at regular intervals of 2–5 weeks, potentially accompanied by aphthous stomatitis, pharyngitis, or cervical adenitis. In addition, written informed consent was obtained from the families. Exclusion criteria included (1) children and families unwilling to participate (e.g., those who strongly requested immediate tonsillectomy), (2) children diagnosed with other serious diseases during follow-up (e.g., one child diagnosed with acute lymphoblastic leukemia), and (3) those lost to follow-up.

In Garavello 2009, 39 children were included. The intervention group had 20 children with a mean age of 5.4 years, and the control group had 19 children with a mean age of 4.9 years. All were early-childhood PFAPA cases, characterized by periodic episodes of acute fever lasting about five days, accompanied by at least one of the following symptoms: aphthous stomatitis, pharyngitis, or cervical lymphadenopathy, without signs of other respiratory infections. The patients responded well to steroid therapy, with symptoms resolving quickly and normal growth between episodes. Patients diagnosed with cyclic neutropenia, other autoinflammatory diseases, immunodeficiencies, or related conditions were excluded.

In Lantto 2024, 16 patients were enrolled. The intervention group consisted of 8 patients with a mean age of 4.5 years, and the control group consisted of 8 patients with a mean age of 3.9 years. Inclusion criteria were: onset of symptoms before 12 years of age, meeting PFAPA syndrome diagnostic criteria (at least five episodes of regularly recurring fever with no other cause, asymptomatic intervals, and normal growth), and written informed consent from the family or legal guardian. Patients requiring concurrent adenoidectomy were excluded.

### PFAPA episodes

In the study by Garavello 2009, the average interval between episodes was approximately 42.4 days in the surgical group and 41.5 days in the control group. The mean duration of each episode was 3.3 days (range: 2–4 days) in the surgical group and 3.5 days (range: 2–5 days) in the control group. The peak fever temperature averaged 39.6 °C (range: 38.7–40.6 °C) in the surgical group and 39.8 °C (range: 38.9–41.0 °C) in the control group. The incidence of aphthous stomatitis was 61% and 58% in the surgical and control groups, respectively, while the incidence of pharyngitis was 98% and 94%, and that of cervical lymphadenopathy was 89% and 82%. None of these differences reached statistical significance (*P* > 0.05).

According to Renko 2007, the average number of episodes per child was 9.0 (range: 5–20) in the tonsillectomy group and 9.5 (range: 4–20) in the control group. The mean duration of each episode was 3.4 days (range: 2–4 days) vs. 3.8 days (range: 2.5–6 days), and the interval between episodes was 25.9 days (range: 21–28 days) vs. 25.0 days (range: 18–28 days). The proportion of children experiencing only fever episodes was 50% in the surgical group and 33% in the control group. The peak fever temperatures averaged 39.7 °C (range: 39.0–40.0 °C) and 39.9 °C (range: 39.0–41.0 °C) in the surgical and control groups, respectively.

Data from Lantto 2024 showed that the mean duration of each individual fever episode was 3.5 days in the tonsillectomy group and 4.2 days in the control group. It should be noted that the values reported in Table 1 for Lantto 2024 (surgical: 0.8 ± 1.4 days; control: 6.5 ± 6.0 days) represent cumulative symptomatic days per person per month (i.e., total fever burden over the follow-up period), rather than the duration of individual episodes. The meta-analysis of fever duration (Figure 4) used this cumulative monthly metric to enable pooling with the comparable outcome from Garavello 2009. The average PFAPA episode interval was 25.6 days in the surgical group and 26.0 days in the control group.

## Summary of the quality assessment

Using the Cochrane Risk of Bias tool (RoB 1.0), three RCTs involving a total of 42 patients who underwent surgery (81 patients in total) were evaluated across all seven domains. A risk-of-bias diagram was generated (Figure 2). All three studies demonstrated certain limitations in mitigating performance bias and detection bias.

Specifically, Renko 2007 did not clearly describe the implementation of blinding, merely stating that “participants were randomly assigned to either the treatment or control group.” Garavello et al. (2009) explicitly noted that “neither patients, parents, nor physicians were blinded to the treatment allocation.” As for Lantto 2024, although a computer-generated random sequence was used, once informed consent for surgery was obtained, the envelope was opened and group assignment was revealed, effectively rendering the study as an open-label design.

## Meta-analysis of the endpoints

### Primary outcome

**Figure 3:**
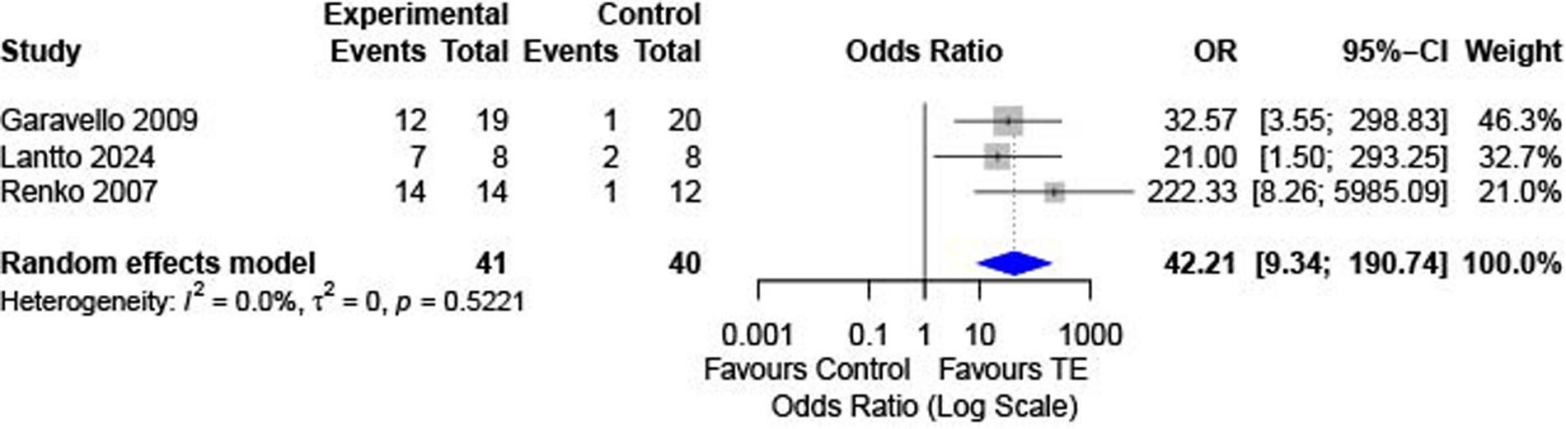
Meta-Analysis of postoperative complete resolution rates in patients with PFAPA syndrome. Effect sizes are presented as odds ratios (OR) plotted on a logarithmic scale; the vertical reference line at OR = 1 indicates no difference between groups.

All three studies included in this analysis reported complete postoperative remission rates in the surgical groups, and these data were summarized in Table 1. A meta-analysis of the three studies' complete remission rates showed that each study favored tonsillectomy (TE) as providing better efficacy for PFAPA patients. The pooled result indicated that patients undergoing surgery had a significantly higher probability of symptom relief compared to controls (pooled OR = 42.21, 95% CI: 9.34–190.74, *P* < 0.0001), with no notable heterogeneity among the studies (I² = 0%, Q = 1.30, *P* = 0.522). Of note, because between-study heterogeneity was estimated as zero (*τ*² = 0), the DerSimonian-Laird random-effects model and the common (fixed)-effect model yield identical pooled estimates; both models are therefore reported for transparency (Figure 3). Although some studies had wide confidence intervals, suggesting small sample sizes, the overall findings suggest that tonsil surgery is associated with a substantially higher probability of complete remission, although the wide confidence intervals reflect the small sample sizes of the included trials. A sensitivity analysis using the Hartung-Knapp-Sidik-Jonkman (HKSJ) adjustment yielded a pooled OR of 42.21 (95% CI: 2.93–609.09, *P* = 0.026), which remained statistically significant (*P* = 0.026). Notably, the HKSJ confidence interval (2.93–609.09) was considerably wider than the standard DL interval, reflecting the increased uncertainty inherent in meta-analyses with very few studies and the more conservative nature of the HKSJ adjustment. The fixed-effect model using the Mantel-Haenszel method produced a consistent estimate (OR = 43.55, 95% CI: 10.01–189.38, *P* < 0.0001). The 95% prediction interval ranged from 9.34 to 190.74, indicating that the true effect in a future comparable study would also be expected to favor surgical treatment. A leave-one-out sensitivity analysis demonstrated that the overall direction of effect favoring surgery was consistent and not driven by any single study: excluding Renko 2007 yielded OR = 27.16 (95% CI: 4.98–148.16, *P* = 0.0001), excluding Garavello 2009 yielded OR = 55.13 (95% CI: 5.67–535.66, *P* = 0.0005), and excluding Lantto 2024 yielded OR = 59.28 (95% CI: 9.43–372.79, *P* < 0.0001), all remaining statistically significant (Table 2). Nevertheless, given the small overall sample size and the limited number of included studies, these results should be interpreted as hypothesis-generating and require confirmation through larger-scale randomized controlled trials (17).

**Table 2:** Leave-one-out sensitivity analysis of postoperative complete remission rates.

Method	Pooled OR	95% CI	*P*-value
DerSimonian-Laird (random-effects)	42.21	9.34–190.74	<0.0001
HKSJ adjustment	42.21	2.93–609.09	0.026
Fixed-effect (Mantel-Haenszel)	43.55	10.01–189.38	<0.0001
Leave-one-out: excluding Renko 2007	27.16	4.98–148.16	0.0001
Leave-one-out: excluding Garavello 2009	55.13	5.67–535.66	0.0005
Leave-one-out: excluding Lantto 2024	59.28	9.43–372.79	<0.0001

### Secondary outcome

Postoperative Complications: None of the three included studies reported postoperative complications such as hemorrhage. However, this should not be interpreted as evidence of surgical safety, as the included trials were small (total *n* = 81), postoperative pain was not systematically assessed in any study, and adverse event reporting may have been incomplete. The absence of reported complications likely reflects limited statistical power to detect uncommon events rather than a true absence of surgical risk.

**Figure 4:**
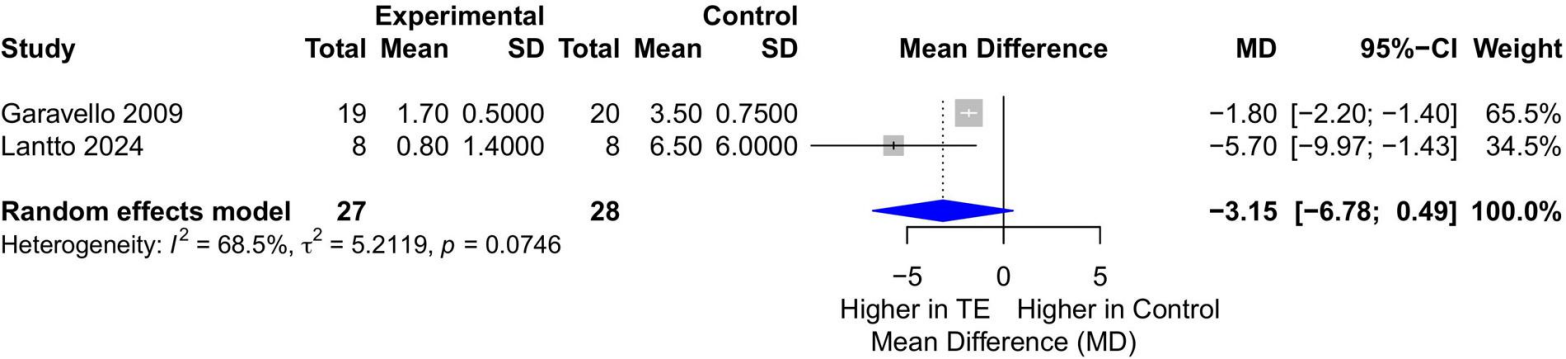
Meta-Analysis of postoperative fever duration: surgical vs. Conservative Treatment.

Duration of Fever Postoperatively: A meta-analysis of the two studies (Garavello 2009 and Lantto 2024) that could be combined indicated that although each study individually suggested that tonsillectomy could shorten the duration of fever episodes (approximately 1.8 days shorter in Garavello 2009 and 5.4 days shorter in Lantto 2024), the pooled mean difference was −3.15 days (95% CI: −6.78 to 0.49), which crossed zero and thus did not reach statistical significance (*P* = 0.090). Substantial heterogeneity (I² = 68.5%) was observed between the two studies, likely reflecting differences in control group medication use (Garavello 2009 permitted glucocorticoids while Lantto 2024 prohibited them) and surgical technique (adenotonsillectomy vs. tonsillotomy). A sensitivity analysis using the HKSJ adjustment yielded a pooled MD of −3.15 days (95% CI: −26.71 to 20.42, *P* = 0.339), with a substantially wider confidence interval that further confirms the statistical uncertainty of this finding. Hence, current evidence does not definitively confirm that tonsillectomy significantly shortens the duration of fever episodes (Figure 4).

**Figure 5:**
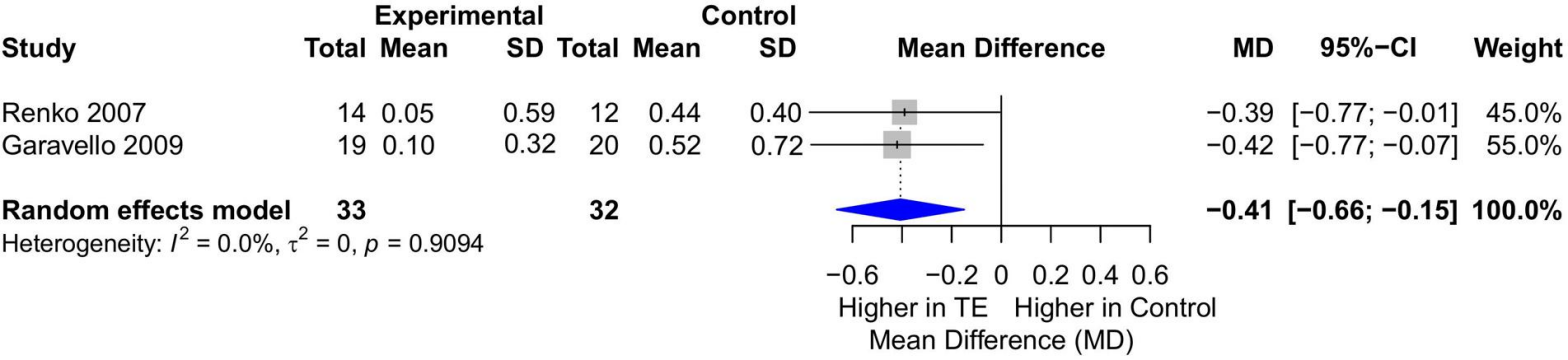
Meta-Analysis of frequency of fever episodes: surgical vs. Conservative Treatment.

Frequency of Fever Episodes: Only Garavello 2009 and Renko 2007 examined changes in attack frequency post-surgery. Renko 2007 demonstrated that after six months of follow-up, children who underwent tonsillectomy experienced significantly fewer episodes (approximately 0.05 episodes/person-month vs. 0.44 episodes/person-month in the control group). In Garavello 2009, the surgical group's average frequency was 0.10 episodes/person-month (SD = 0.32) compared to 0.52 episodes/person-month (SD = 0.72) in the control group. The pooled mean difference was −0.41 episodes/person-month (95% CI: −0.66 to −0.15, *P* = 0.002), with no heterogeneity (I² = 0%). Notably, no patients in the surgical group experienced another episode after 12 months of follow-up (Figure 5). A sensitivity analysis with the HKSJ adjustment confirmed the robustness of this result (pooled MD = −0.41 episodes/person-month, 95% CI: −0.60 to −0.22, *P* = 0.023), supporting a statistically significant reduction in episode frequency following tonsillectomy.

Medication Use During Follow-Up: The three studies varied regarding medication use during follow-up. In Garavello 2009 and Renko 2007, both the surgical and control groups were allowed to use corticosteroids (e.g., prednisone) to briefly alleviate symptoms during follow-up, although some patients still ultimately required surgical intervention. Garavello 2009 reported that 50% of the surgical group and 88% of the control group used corticosteroids during the follow-up period, but details on dosage and frequency were not provided. In contrast, Lantto 2024 explicitly stated that neither group received corticosteroids during follow-up; the control group relied on the natural progression of the condition or eventually underwent a rescue tonsillectomy.

Overall, these findings suggest that tonsil surgery (encompassing tonsillectomy, adenotonsillectomy, and tonsillotomy) may provide improvement in PFAPA symptoms compared to conservative management alone, although the clinical heterogeneity among surgical approaches and the small number of included studies warrant cautious interpretation of the pooled estimates.

## Discussion

This meta-analysis, synthesizing existing research evidence, demonstrates that children with Periodic Fever, Aphthous Stomatitis, Pharyngitis, and Adenitis (PFAPA) who undergo tonsillectomy show significantly better improvement in core symptoms compared to those receiving conservative treatment, though the strength of evidence varies across different dimensions. Meta-analysis reveals that the post-operative complete remission rate in the surgical group was significantly higher than the control group (OR = 42.21, 95% CI: 9.34–190.74, *P* < 0.0001), with extremely low heterogeneity (I² = 0%) among the three included randomized controlled trials (RCTs). This finding was confirmed by sensitivity analyses using the more conservative HKSJ method, which also yielded a statistically significant result, and was robust in leave-one-out analysis, lending additional support to this finding, although the small number of included studies limits definitive conclusions. While these findings are encouraging, their clinical implications should be interpreted cautiously given the limited evidence base. For children with refractory PFAPA (meeting modified Marshall diagnostic criteria with frequent episodes persisting despite conservative management), tonsil surgery may be considered as a treatment option, particularly for those experiencing diminishing response to glucocorticoid treatment or facing significant medication side effects. However, given that PFAPA is a self-limiting condition that typically resolves spontaneously within several years, the decision to proceed with surgery must be carefully individualized, weighing the potential benefits of earlier symptom resolution against surgical risks and the likelihood of spontaneous remission. Notably, Lantto's 2024 study, through a trial design completely discontinuing medication intervention, demonstrated that the surgical group achieved an 88% nine-month relapse-free survival rate without any concurrent medication, providing additional evidence for evaluating the independent impact of surgery on the natural disease course, although the small sample size (*n* = 16) limits the generalizability of this finding.

However, conclusions regarding fever duration and episode frequency remain relatively less definitive for certain outcomes, as further explored by the HKSJ sensitivity analyses. For fever duration, substantial heterogeneity was observed (I² = 68.5%, Q = 3.18, *P* = 0.075), likely attributable to differences in medication protocols and surgical techniques between studies, and the HKSJ adjustment produced a markedly wider confidence interval (MD = −3.15, 95% CI: −26.71 to 20.42, *P* = 0.339), indicating considerable statistical uncertainty. In contrast, for episode frequency, the HKSJ analysis confirmed a significant reduction favoring surgery (MD = −0.41, 95% CI: −0.60 to −0.22, *P* = 0.023), supporting the robustness of this finding. Furthermore, the included studies differ in research design, blinding implementation, and subject enrollment criteria, leading to certain degrees of bias and heterogeneity. Regarding fever frequency, both Garavello 2009 and Renko 2007 addressed this issue, with both studies supporting the superiority of surgical treatment over conservative management in reducing post-operative episode frequency. However, both studies allowed the control group to use glucocorticoids as needed during follow-up, while Feder's study suggests that frequent steroid use may “artificially increase” the attack frequency in the conservative group by shortening the inter-episode interval (23). Additionally, in Garavello's study, 15% of patients in the surgical group still required intermittent steroid use to control residual symptoms post-operation (average 0.8 times/month), suggesting that tonsillectomy is not an absolute curative treatment and may work synergistically with medication. Importantly, this observation raises a methodological concern: if a substantial proportion of the surgical group continued to receive corticosteroids post-operatively, the observed benefit may reflect a combined surgical-medical effect rather than the independent efficacy of surgery alone. This potential confounding should be considered when interpreting the magnitude of surgical benefit reported in these studies.

Previous studies have shown that tonsil surgery can quickly reduce the impact of PFAPA on families and improve children's quality of life; in its 2011 and 2019 guidelines, the American Academy of Otolaryngology-Head and Neck Surgery included PFAPA as one of the optional indications for tonsillectomy (24, 25). When considering surgical intervention, it is crucial to thoroughly weigh the associated risks against potential benefits. Tonsillectomy, while generally safe, carries inherent surgical risks including peri-operative bleeding (estimated 1% for primary hemorrhage), anesthesia-related complications, postoperative pain affecting oral intake, and rare but serious infections (24, 26). These risks must be carefully contextualized against the natural history of PFAPA, which typically follows a self-limiting course with spontaneous resolution in many cases within several years.

The clinical decision-making process should therefore incorporate multiple factors: the frequency and severity of PFAPA episodes, the degree of life impairment for both child and family, response to previous medical treatments, and the family's preference regarding surgical intervention vs. continued medical management. For children with frequent, severe episodes significantly affecting school attendance, family functioning, or growth development—particularly those showing poor response to glucocorticoids or experiencing substantial medication side effects—tonsillectomy may offer substantial benefits that outweigh the surgical risks. Conversely, for children with milder symptoms or those closer to the typical age of spontaneous resolution, a conservative approach with watchful waiting may be more appropriate. Clinical decisions require doctors to combine each child's actual situation, fully communicate with parents about both surgical risks and expected benefits, and make the most beneficial choice for the child between surgery and waiting for natural recovery.

The meta-analysis results in this paper have the following limitations: 1. This systematic review was not prospectively registered in PROSPERO or any other systematic review registry. Although the restriction to a small number of pre-identified RCTs (*n* = 3) somewhat limits the risk of outcome-reporting bias, the absence of prospective registration should be acknowledged as a limitation; 2. Only three RCTs with a total of 81 patients met the inclusion criteria, and for secondary outcomes, only two studies could be pooled. As highlighted by Zhou and Shen (17), meta-analyses with few studies (<=5) and small sample sizes (<30 patients per group) face inherent statistical challenges: the I² statistic for assessing heterogeneity is biased and imprecisely estimated, and standard random-effects methods such as the DerSimonian-Laird approach may produce unreliable estimates with inflated Type I error rates. Although we performed sensitivity analyses using the HKSJ adjustment, prediction intervals, and leave-one-out analysis, all of which supported the primary conclusion, the overall results should be interpreted with caution and considered hypothesis-generating rather than definitive; 3. Most studies failed to achieve double-blind design, and subjective judgments from patients and evaluators may lead to performance bias and detection bias; 4. While surgery promotes remission, the evidence offers little guidance for weighing this benefit against the self-limiting natural history in individual patients, hindering personalized risk-benefit assessments; 5. Inconsistent medication interventions during follow-up (with Garavello 2009 and Renko 2007 permitting glucocorticoid use while Lantto 2024 prohibited it) may confound the evaluation of surgical efficacy; 6. The three included studies employed different surgical techniques (tonsillectomy in Renko 2007, adenotonsillectomy in Garavello 2009, and tonsillotomy via Coblation in Lantto 2024), which introduces clinical heterogeneity. The limited number of studies precludes meaningful comparison between specific surgical approaches (e.g., via network meta-analysis), and future research with standardized surgical protocols and larger sample sizes is needed to determine whether certain techniques offer superior outcomes; 7. The exclusion of non-English language publications may have introduced language bias, potentially omitting relevant studies published in other languages; 8. Because the search was limited to three databases, relevant studies indexed elsewhere may have been missed. Furthermore, although quality of life (QoL) is explicitly identified in the Introduction as a major concern for PFAPA patients and their families, no QoL outcome could be included in this meta-analysis due to the absence of standardized QoL data in the included RCTs. This represents a notable gap, and future studies should incorporate validated QoL instruments to capture the broader impact of surgical intervention, as suggested by Rydenman et al. Future research should focus not only on efficacy but also on developing predictive models to stratify patients, ideally identifying those for whom surgery is unequivocally indicated vs. those for whom watchful waiting remains the most favorable option.

## Conclusion

This meta-analysis suggests that tonsillectomy may provide benefits for children with PFAPA, especially those who fail to improve under conservative management. Our findings indicate a notably higher complete remission rate in surgical groups, a conclusion supported by sensitivity analyses using the more conservative HKSJ method, prediction intervals, and leave-one-out analysis. However, evidence regarding fever duration remains inconclusive due to substantial heterogeneity and statistical uncertainty, whereas the evidence for reduced episode frequency is more robust, having been confirmed by HKSJ sensitivity analysis (*P* = 0.023). Additionally, the quality and quantity of available studies are limited, with variation in design, blinding, medication use, and surgical techniques. Given the small number of included studies and sample sizes, these results should be considered hypothesis-generating rather than definitive (17). The pooled estimates should be interpreted cautiously given the small number of trials and the clinical heterogeneity across surgical approaches. There is also a lack of data on postoperative complications and pain. Future large-scale, well-designed RCTs with standardized surgical protocols are needed to confirm these results, refine treatment protocols, compare specific surgical techniques, and ensure safer, more effective management of PFAPA.
